# Hcp/fcc nucleation in bcc iron under different anisotropic compressions at high strain rate: Molecular dynamics study

**DOI:** 10.1038/s41598-018-25758-1

**Published:** 2018-05-16

**Authors:** Jian-Li Shao, Pei Wang, Feng-Guo Zhang, An-Min He

**Affiliations:** 1 State Key Laboratory of Explosion Science and Technology, Beijing Institute of Technology, Beijing, 100081 China; 2 Institute of Applied Physics and Computational Mathematics, Beijing, 100094 China

## Abstract

Previous researches have revealed the importance of shear and the orientation dependence in the structural transition of iron. In this work, we introduce a series of shear deformations by adjusting the strain ratio between the longitudinal ([001]) and transversal ([010] and [100]) directions, and then investigate this structural transition under different anisotropic compressions with molecular dynamics simulations. It is found that the shear deformation can lower the transition pressure notably, and even change the nucleation structure and morphology. Under 1D-dominated compression (along (001) direction), there only appears hcp nucleation with a few fcc stacking faults. For other cases, more equivalent planes will be activated and fcc structure begins to nucleate. Under 2D-dominated compression (along (010) and (001) directions), the fcc mass fraction is already over the hcp phase. At last, we compare the variations of shear stress and potential energy for different phases, and present the sliding mechanism under typical anisotropic compressions.

## Introduction

Most metal crystals may experience a polymorphic transition under shock compressions, which belongs to the first order phase transition from a thermodynamic point of view. This kind of phase transition can essentially change the physical and dynamic properties of materials. Iron is a prototypical metal of structural transition and it experiences the bcc to hcp transition at pressure of about 13 GPa. This phase transition was first discovered by Bancroft *et al*.^1^ in the shock wave experiments, and later experiments identified it as the bcc to hcp phase transition by using x-ray diffraction methods^2,3^. With the abundant technological applications, a comprehensive understanding of the bcc to hcp transition in iron is of great significance. To this day, the physical mechanism underlying this transition and its dynamic characteristics have been explored intensively and extensively in theoretical^4–11^ and experimental^12–19^ researches.

As we know, the phase transition is essentially a multi-scale problem, which originates from the atomic-scale and propagates to the macro-scale. In other words, the transition pressure and structures are strongly affected by the microscopic constituents of materials. Thus, the theoretical modelling based on macroscopic views will encounter many difficulties when involving the nucleation dynamics. Fortunately, with the rapid development of the computer capability, molecular dynamics (MD) simulation based on the atomic interaction has been a powerful tool to explore the microscopic mechanism of phase transition even though its simulation size is still limited. And Significant progress has been made in studies of the shock structural transition and related mechanism. Such as single crystal^20,21^ and polycrystal^22^, uniaxial^23–25^ and uniform^26^ compressions, homogeneous^26,27^ and heterogeneous^28,29^ nucleations, plasticity and phase transition^30–32^.

Among them, the bcc to hcp transition in iron under shock compression was firstly simulated by Kadau *et al*.^20^, and the slip mechanism suggested by MD simulations was confirmed by Kalantar *et al*. via ultra-fast *in situ* x-ray diffraction studies of iron^14^. Besides, the hcp shape determined by the experiment^15^ is also similar to the MD results, in the size range between 2 and 15 nm. Later, the effects of voids on that structural transition under shock compression was investigated^28,29^, where the void was found to reduce the threshold pressure and accelerate the nucleation speed. Furthermore, the atomic mechanical path shows that the transformed atoms do cross a shear pressure barrier and then show an over-relaxation of pressure^29^. In addition, the polycrystal simulations were also carried out^22^, and the Hugoniot curves are found more close to the experiments.

However, as reported early by shock experiment^33^, the critical pressure of the structural transition is still difficult to be forecasted accurately. In fact, previous MD simulations have shown the importance of shear deformation. For an example, the bcc-fcc transition were found under shock^21^ or high strain rate^24^ compressions along the [011] and [111] directions. Of course, that remains to be confirmed by the experiment. Also some studies^7,34^ have revealed the importance of shear stress on the bcc to hcp transition in iron through a multi-scale model analysis.

In the present work, the high strain rate compression is used to approximately simulate the shock loading. And then, we introduce a series of shear deformations by adjusting the strain ratio along the three compression directions. So that the influence of shear deformation is analyzed systematically with MD simulations. And our simulations are all performed at a high strain rate compression Our results demonstrate the influence of shear on the nucleation pressure, nucleation structure, and also dynamic properties for different phases. The rest of this paper is organized as follows. Section II briefly describes the computational details, including the potential model, strain control and some analysis skills. In Sec. III, we present our results and discussion on the dynamic and microscopic processes. Our main conclusions are summarized in Sec. IV.

## Computational Details

The interaction between atoms is described by the embedded-atom-method (EAM) potential developed by Voter-Chen^35^, for which can well reproduce the bcc to hcp/fcc structural transition of iron^20–22,25^. The initial configuration consisting of 80_*x*_ × 80_*y*_ × 80_*z*_ bcc unit cells, where the *x*, *y* and *z* axes are oriented along the [100], [010] and [001] directions respectively. And the periodical boundary condition is employed in all the three directions.

After a sufficient relaxation of the sample at the temperature of 60 K in the canonical ensemble, we perform the simulations on the anisotropic compression. It is known that the strain constraint maybe have a distinct effect on the structural transition. Here, we just consider the case that bulk strain *ε*_*b*_ is allocated to the three directions as following: *a*_*x*_ = *a*_0_(1 − *ε*_*b*_*s*), ${a}_{y}={a}_{z}={a}_{0}(\sqrt{\mathrm{(1}-{\varepsilon }_{b}\mathrm{)/(1}-{\varepsilon }_{b}s)})$. *a*_0_ is the initial lattice constant (2.8725 Å), and *a*_*x*,*y*,*z*_ are the variables along the three directions during compression. Thus, different shear states can be obtained by varying the adjustable parameter *s* from 1 (1D compression) to 0 (2D compression), and then the compression can be achieved by contracting the lattice constant *a*. For all the loading paths, the NVE ensemble is used and the bulk strain rate is fixed at the same value (1.5 × 10^9^ s^−1^). And all the simulations are performed with the MD code developed by ourselves. As for other arbitrary shear strains, the simulation results will contain more possibilities, for which we need to further explore the strain factor so as to perform suitable simulations.

Based on the simulation results, we discuss both the dynamic properties and microstructures in detail. The normal stresses are calculated according to the virial formula^36,37^ and the other physical quantities (temperature and potential) are calculated by averaging. Firstly, the structural transition is shown by the radial distribution functions (RDFs), and then the hcp/fcc close-packed atoms are identified by their respective coordination numbers (CNs), where the cut-off radius are determined by the first and second peaks of RDF. Further, the hcp or fcc structures are distinguished from each other by the centro-symmetry parameter (CSP)^38^.

## Results and Discussion

### Dynamic properties

**Figure 1:**
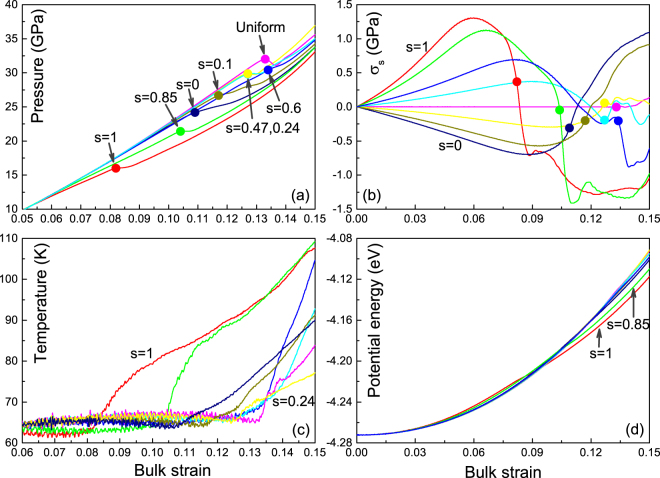
Variations of pressure (**a**), shear stress *σ*_*s*_ (**b**), temperature (**c**) and potential energy (**d**) with the bulk strain. The pressure threshold of the structural transition (solid circles) is found to vary markedly with the value of *s*. Accordingly, the over-relaxation of shear stress and the increase of temperature and potential energy are shown.

Above of all, a significant influence of shear deformation on the structural transition in iron is revealed. In Fig. 1, we present the variations of pressure, shear stress, temperature and potential energy with bulk strain under different shear deformations. Here, the pressure is the mean value of the three normal stresses. And for convenience, we calculate the maximal shear stress using the formula ${\sigma }_{s}=\frac{1}{2}\times ({\sigma }_{z}-\frac{1}{2}\times ({\sigma }_{x}+{\sigma }_{y}))$. In fact, the shear stresses can also be calculated directly using virial formula. And the results are found to be equivalent. Like other simulations^20–22^, the structural transition is captured with increasing the compression strain. One can see the pressure peaks or inflection points in Fig. 1(a), which indicate the onset of structural transition and their values can be thought as the thresholds. It is found that the threshold increases gradually from ∼16 GPa (*s* = 1, 1D compression) to ∼32 GPa (uniform compression). With further reducing the value of *s*, the sample enters into a 2D-dominated compression states and the pressure threshold begins to reduce again, except that the peak pressure at *s* = 0.6 is somewhat higher than at *s* = 0.47. The relaxation of shear stress is observed prior to structural transition (Fig. 1(b)) owing to the softening of elastic modulus in the compressive direction. As the structural transition goes on, shear stresses will get into an over-relaxation state, lasting for a certain strain interval, especially for the cases of lager shear deformation. This was also mentioned in the previous simulations on shock-induced structural transition^21^. There is also a distinct heat release during the structural transition for all the cases, shown in Fig. 1(c), so the nucleation of new phase is factually reflected on the temperature curves. It seems that the cases of *s* = 0.6 and *s* = 0.47 have a faster temperature rise thanks to the reduction of *σ*_*s*_. Note that, the initial temperature is set at 60 K in order to obtain a clear microstructure without a marked heat effect. In this case, the structural transition will not loss the generality with temperature, except that the transition pressure at 60 K is slightly higher than 300 K. And as for the temperature rise, it is more distinct at lower temperature. The changes of potential energy (Fig. 1(d)) show that: before the structural transition, the shear deformation enhances potential energy of the sample (especially for the cases *s* = 1 and *s* = 0.85); while after structural transition, the shear deformation makes the potential energy of the sample lower.

**Figure 2:**
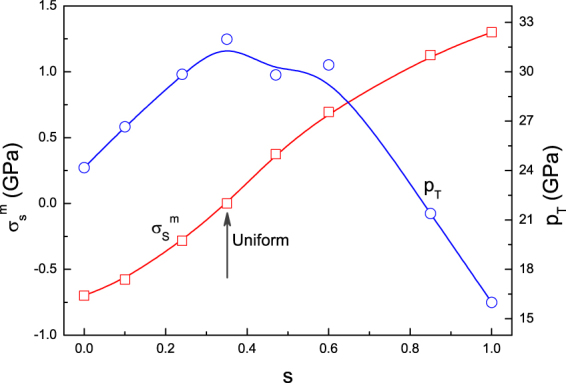
Pressure threshold *P*_*T*_ of the structural transition and shear stress peak ${\sigma }_{s}^{m}$ before the structural transition as functions of *s*. *P*_*T*_ shows an approximate linear decrease with the increase of the ${\sigma }_{s}^{m}$ magnitude.

From the above pressure and shear stress curves, we extract all the pressure thresholds (*P*_*T*_) and the shear stress peaks (maximum or minimum, ${\sigma }_{s}^{m}$), which are presented in Fig. 2. It can be seen that ${\sigma }_{s}^{m}$ decreases from 1.3 GPa to −0.7 GPa as *s* reduces from 1 to 0, and this variation is approximately linear. The pressure threshold *P*_*T*_ is found to arrive at its maximum under uniform compression, and show a linear decrease with increasing of the magnitude of ${\sigma }_{s}^{m}$. In short, under the anisotropic compression we set, the transition threshold keeps an opposite change with the increase of shear deformation. This change law is based on the given loading pathes covering the three typical compressions. And for arbitrary shear conditions, the shear effects is still to be examined by developing an effective loading method.

### Hcp/fcc nucleation and growth

**Figure 3:**
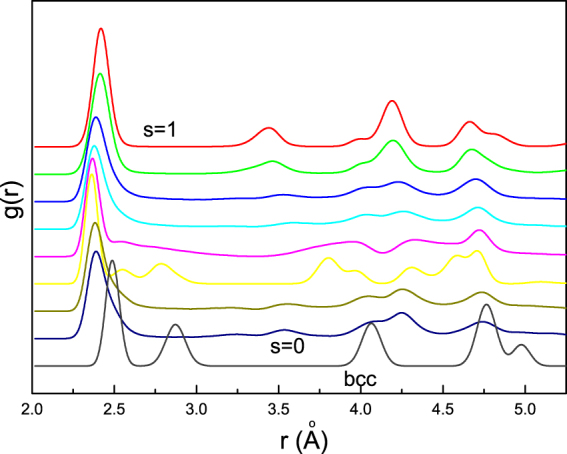
Radial distribution functions for different loading paths at the bulk strain of 0.15. The combination of the first two peaks (except the case of *s* = 0.24, shown by yellow line) can be seen, which indicates the formation of the close-packed structures.

Obviously, the above statistical characteristics essentially reflect the microstructure difference during the structural transition. Note that plastic deformation never happens before structural transition for the potential used here. So we can conveniently give the RDF analysis on the structural transition. The RDF results corresponding to the whole model is shown in Fig. 3. We can see that, the perfect lattice of bcc iron has four distinct RDF peaks in the range *r* < 5.5 nm (the gray line). With increasing the compression strain, we can observe the shift leftwards or split of the peaks. When the strain arrives at the critical value, a certain number of the next-nearest neighbor atoms will turn into the nearest and hcp/fcc structures start nucleating, and others move outward from their original positions. So that the first two peaks merge into one peak accompanied with the formation of some new peaks for all the cases but *s* = 0.24, where the first two peaks doesn’t merge because the slip amplitude of atoms is much smaller than other cases.

**Figure 4:**
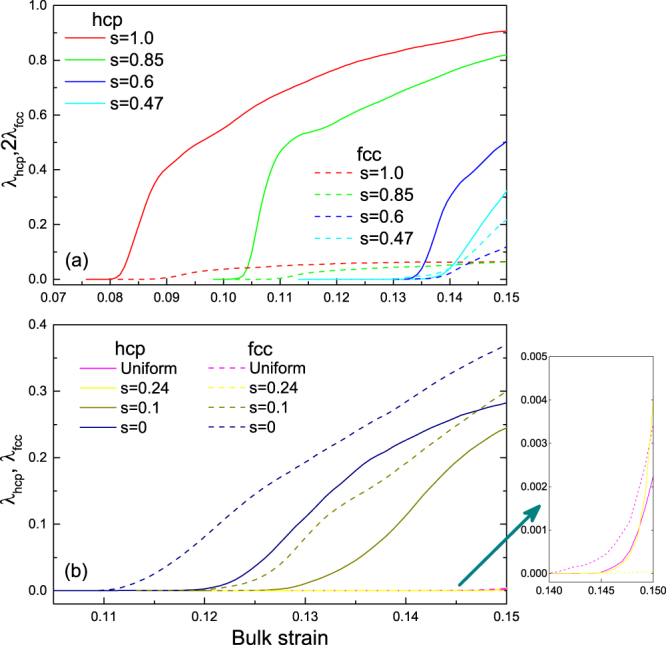
Mass fractions *λ*_*hcp*_ and *λ*_*fcc*_ versus bulk strain for different values of *s*. For the cases of *s* = 0 0.85 and 1, there mainly form hcp structures after structural transition. With the reduction of *s*, hcp structures decrease and meanwhile fcc structures increase.

Now we explore the hcp/fcc nucleation and growth. The variation of mass fractions (*λ*_*hcp*_ and *λ*_*fcc*_) under different anisotropic compressions is presented in Fig. 4. Here, the close-packed atoms are identified by their respective coordination numbers, and the cutoff distance is fixed at 0.265 nm in view of the above RDFs. Further, hcp atoms are extracted from the close-packed atoms by CSP > 0.35 Å. In the cases of *s* > 0.6, the results of *λ*_*hcp*_ and *λ*_*fcc*_ indicate that hcp nucleation plays a dominant role, while fcc structures only exit as stacking faults. With reducing the value of *s*, we find the fcc nucleation though *λ*_*fcc*_ is quite less than *λ*_*hcp*_. However, under 2D-dominated compressions, *λ*_*fcc*_ increases rapidly and even over *λ*_*hcp*_. Of course, the mass fraction lies on the method and parameters to some extent, but this is thought not to change the scaling behavior.

**Figure 5:**
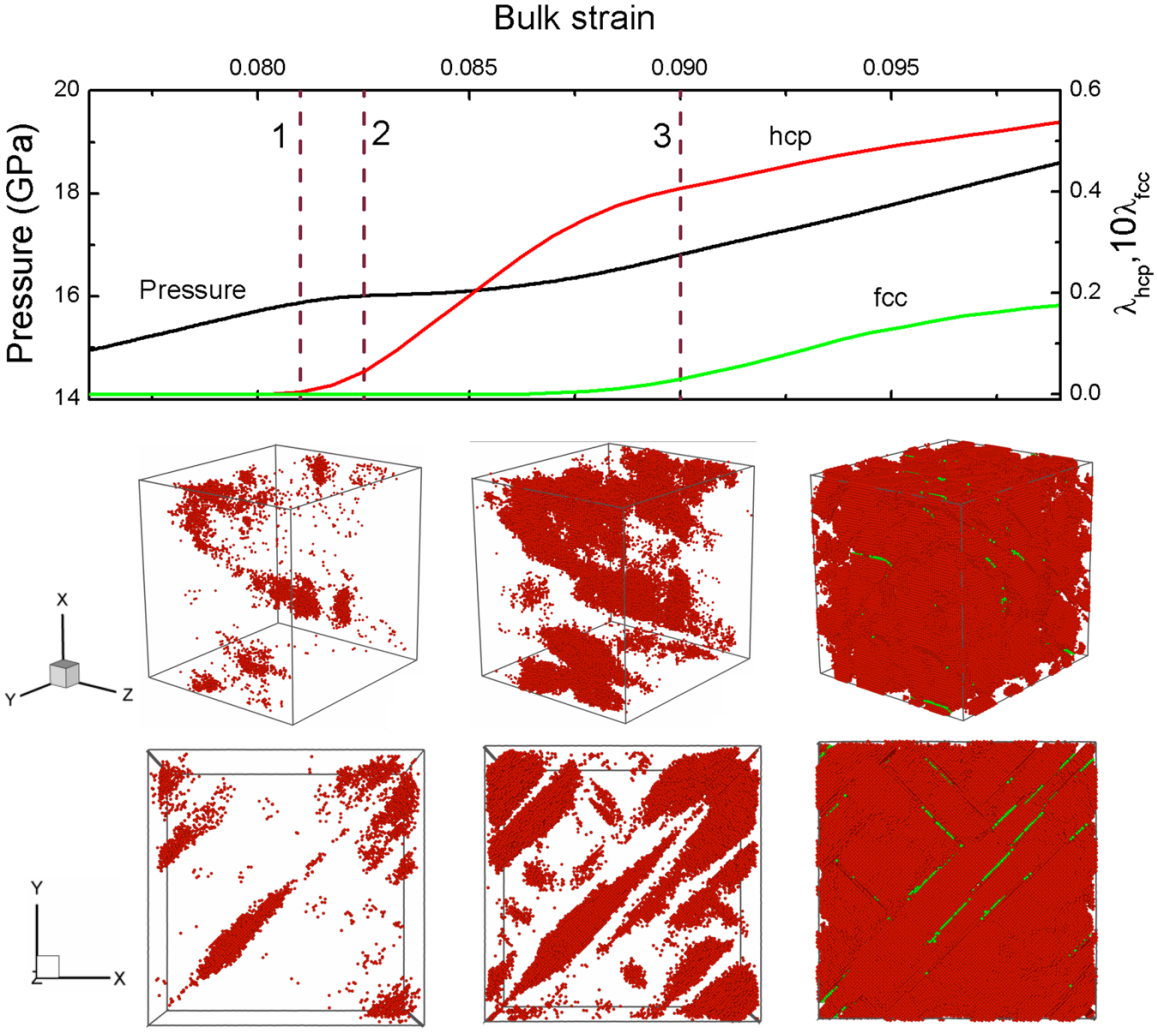
Variations of the pressure and mass fraction of structural transition with bulk strain for *s* = 1, and the microscopic views of the hcp/fcc nucleation and growth. Red and green atoms correspond to hcp and fcc structures respectively, and other atoms are removed.

**Figure 6:**
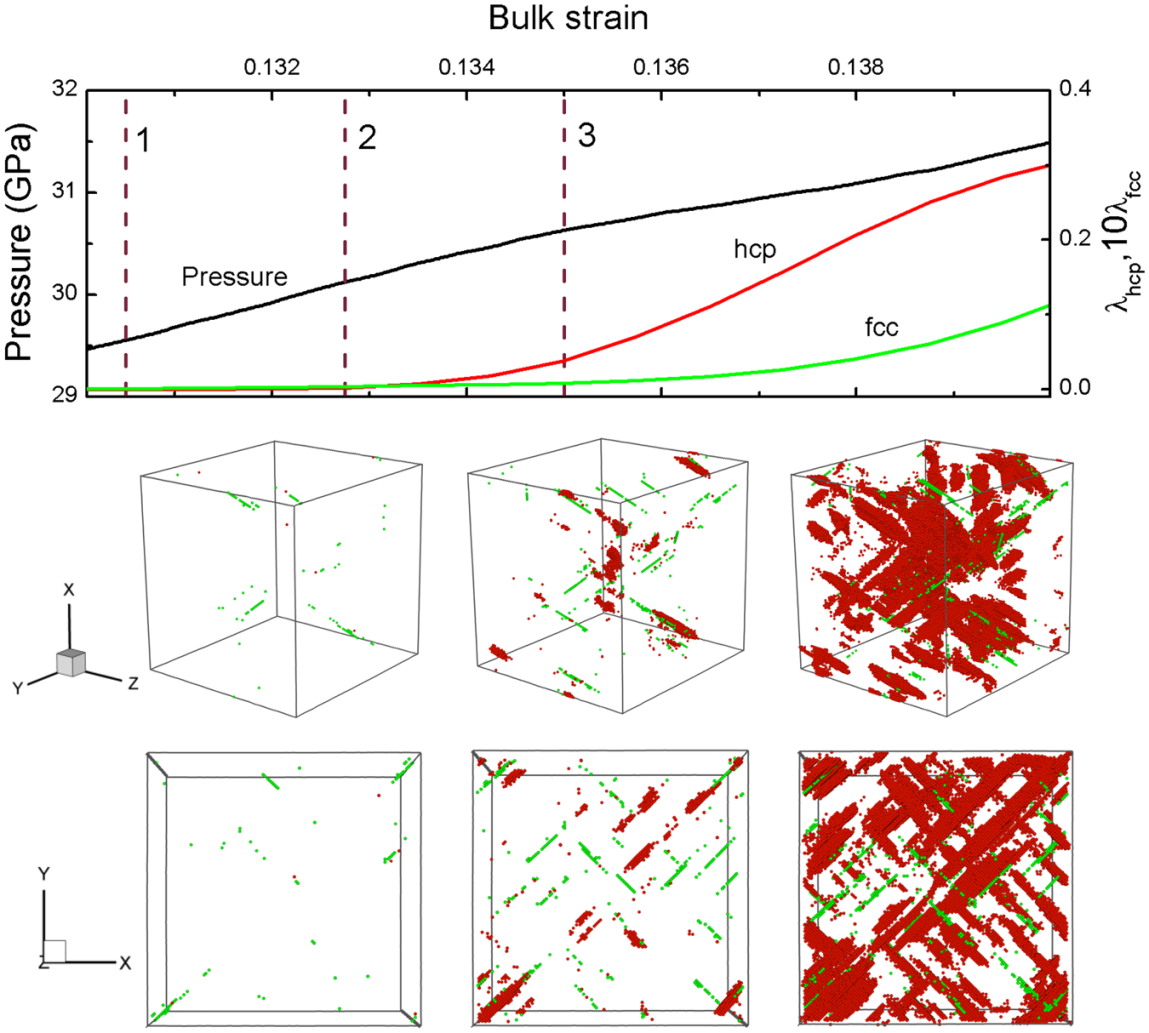
Variations of the pressure and mass fraction of structural transition with bulk strain for *s* = 0.47, and the microscopic views of the hcp/fcc nucleation and growth. Color coding as in Fig. 5.

**Figure 7:**
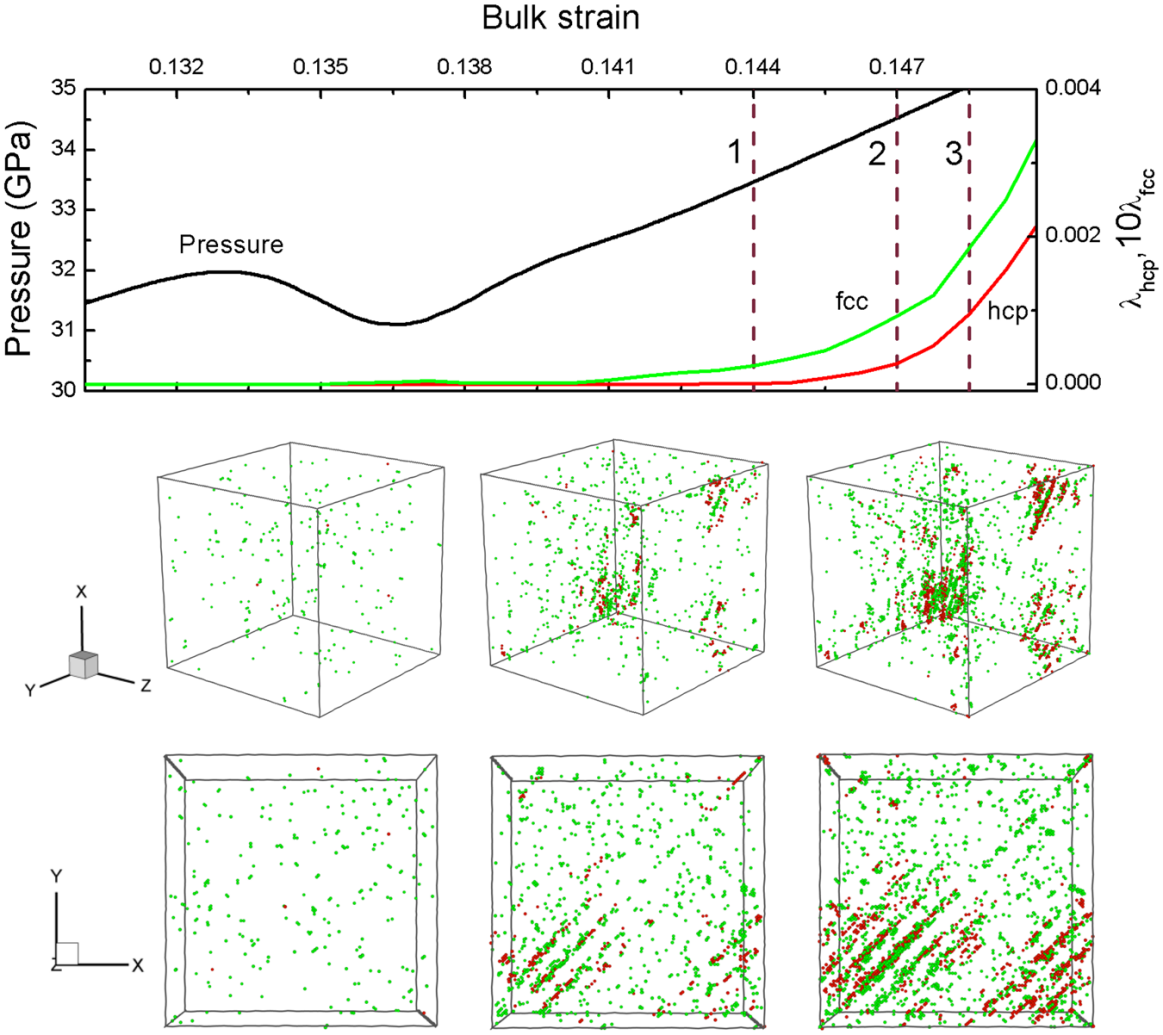
Variations of the pressure and mass fraction of structural transition with bulk strain under uniform compression, and the microscopic views of the hcp/fcc nucleation and growth. Color coding as in Fig. 5.

**Figure 8:**
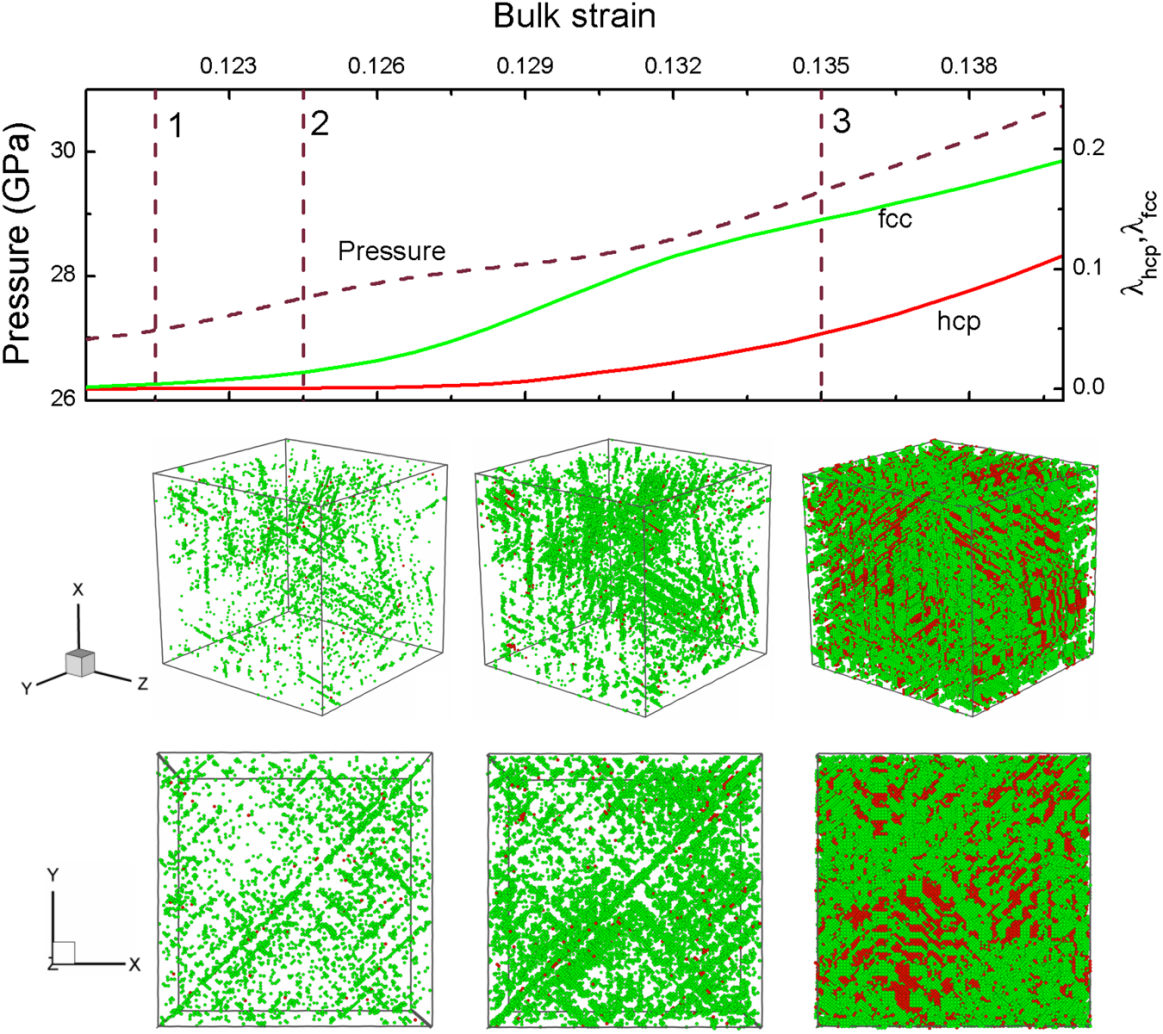
Variations of the pressure and mass fraction of structural transition with bulk strain for *s* = 0.1, and the microscopic views of the hcp/fcc nucleation and growth. Color coding as in Fig. 5.

Figures 5–8 show respectively the microscopic views of hcp/fcc nucleation and growth under typical shear deformations. As shown in Fig. 5, we find hcp nucleation occurs from 1 to 2 in the case of *s* = 1, and accordingly, the pressure shows an inflection (∼16 GPa) in this interval. Subsequently, there is a quick growth of new phase from 2 to 3. And these hcp nuclei will grow into flaky morphology along (110) and (1–10) planes. But, fcc structure can only form as stacking faults, wherever the two hcp grains meet each other at the same invariant planes.

With the increase of *s*, the fcc nucleation is definitely to happen. For instance, Fig. 6 shows the microscopic views of both hcp and fcc nucleations for the case of *s* = 0.47. In this case, a few fcc structures are originally observed, and then both fcc and hcp structures begin to nucleate along 〈111〉 directions. Nevertheless, the growth speed of hcp nucleus is much faster than that of fcc nucleus. Finally, these new crystal nuclei can grow into lath morphology along all the {110} planes, and the grains of new phase are smaller than those in Fig. 5.

The uniform compression corresponds to the highest transition pressure. In this case, all the potential slip planes are exactly equivalent, which increases the difficulty of hcp/fcc nucleation because of no more preferential slip plane. As shown in Fig. 7, there is a distinct inflection of pressure curve prior to apparent hcp/fcc nucleation, where the pressure reduces to ∼31 GPa from ∼32 GPa. We can only observe many minute hcp/fcc embryos or clusters before the strain 1. As the compression strain arrives at strain 2, there begin to form some small hcp/fcc nuclei along the {111} directions.

While under 2D-dominated compressions, the fcc nucleation already plays an important role, and even over the hcp nucleation. In Fig. 8, we can see many fcc nucleations come into being along {111} from strain 1 to 2, and then some hcp structures begin to nucleate. All these hcp/fcc nuclei grow into slender rod shape, and finally there form many grains consisting of alternate hcp and fcc structures. In fact, the hcp and fcc nuclii may coalesce into a flaky shape along the equivalent {110} except (110) and (1–10) planes.

### Microscopic processes

**Figure 9:**
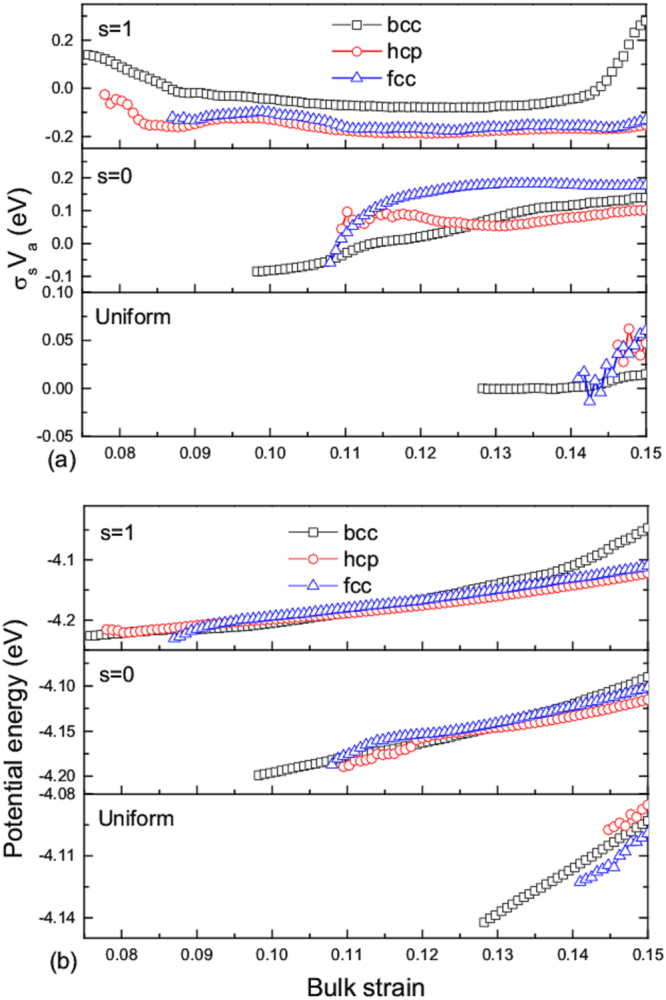
Variations of shear pressure-volume *τV*_*a*_ and potential energy for the cases of *s* = 1, *s* = 0 and uniform compression with bulk strain. The over-relaxation for the cases of *s* = 1 and *s* = 0, and the production of shear stress for uniform compression are shown. In the mixed phases, the potential energy of hcp/fcc structures will become lower than bcc structure after their early nucleations (see the cases of *s* = 1, and *s* = 0).

In order to further understand the transition dynamics, we compare different microscopic paths under the three typical compressions, which are the uniform, 1D ([001]) and 2D ([100] and [010]). There is no doubt that the phase transition occurs because the free energy of the new phase is lower. The nucleation of phase change can make the system stress relax, but the phase transformation can only be partially completed under the certain strain constraint. Thus, the mix phases appear in the system. Here, the variations of the phase energy and shear stress in the mixed phase are shown in Fig. 9(a) and (b), respectively. The simulation results show that both hcp and fcc are in a relatively low energy state under the 1D and 2D compressions. Under 1D compression, at the beginning of nucleation, we find the hcp structure and also fcc stacking fault are already in an over-relaxation of shear stress. And the hcp potential energy becomes lower than bcc structure as they continuously grow because of the strain relaxation. Under 2D compressions, phase transition can indeed be more prone to metastable fcc structure although the energy of hcp structure is much lower, which is obviously attributed to high strain rate and strain constraint. Similarly, we observe that the shear stresses turn into positive value after the transition under 2D compression, also showing an over-relaxation state. And the fcc structure can relax to the highest shear stress, which maybe result in that the mass fraction *λ*_*fcc*_ is over *λ*_*hcp*_. Although the hcp potential energy is a little lower than fcc structure. Under uniform compression, the system can also produce some shear stress after the hcp/fcc nucleation. However, the initial nucleation volume is too small and the hcp particles exhibit the higher interfacial energy, which is certainly affected by statistical methods to some extent. All these result further illustrate the important effect of shear stress on the nucleation of new phase. And from these results, we can obtain that hcp structure is directly formed only under the direction of compression along the direction of [001], and the hcp and fcc structures will be formed under other strain conditions, as for this potential we used. These results are still to be tested and further analysis will be performed for the static loading case and different potentials later.

**Figure 10:**
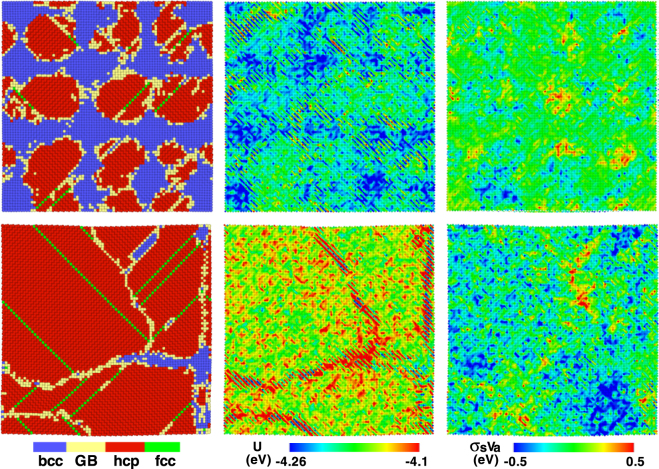
Hcp nucleation and growth in the case of *s* = 1, and the corresponding distributions of shear pressure-volume *τV*_*a*_ and potential energy.

Moreover, the distributions of shear stress and potential energy during the transition process under 1D compression are presented in Fig. 10. We can clearly see the partition of shear stress and potential energy with appearance of new structures. At the early nucleation stage, the hcp potential energy is higher than the bcc structure, and a mount of hcp atoms are in over-relaxation state. Obviously, these are consistent with the above statistical results. When the whole sample finishes the structural transition, there form some grain boundaries composed of alternate higher or lower potential atoms. And most hcp structures are already in over-relaxation state.

**Figure 11:**
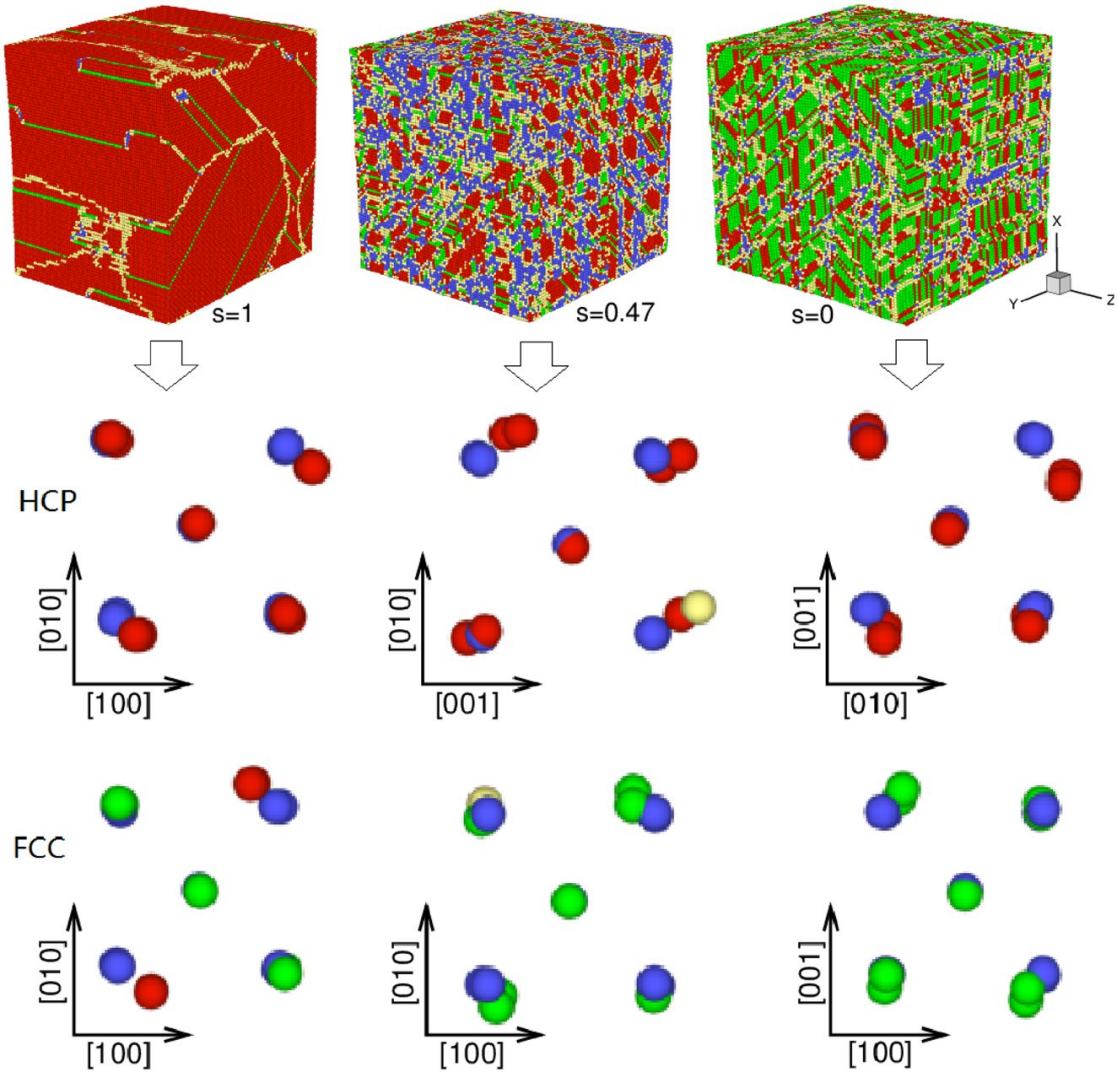
(Upper) Microscopic views in the cases of *s* = 1, *s* = 0.47 and *s* = 0, respectively. The grain structure and size changes can be seen clearly. (Lower) The corresponding sliding mechanism of atoms, where the blue atoms represent the initial bcc unit cell.

Figure 11 presents the final morphology after structural transition and the sliding mechanism of atoms for some typical processes. Obviously, there form larger hcp grains running through *z* axis under 1D compression (*s* = 1). In this case, the slip mechanism of hcp formation is the relative shuffle of adjacent (110) or (1–10) faces along the^1–10^ or [110] direction, consistent with the simulations under shock loading^20^. And if the two adjacent (110) or $(1\bar{1}0)$ faces shuffle in opposite directions, then fcc structure forms. In the case of *s* = 0.47, nearly all the equivalent slip systems are activated and accordingly the new phases grow into smaller grains. Besides, some flaky twins along the {110} planes are observed. Under 2D compression (*s* = 0), the fcc nucleation begins to play an important role and its mass fraction is even over hcp phase. The hcp-fcc mixed phases also take on flaky grains along equivalent {110} planes. And finally many complex flaky twins take shape due to intercrossing of the different flaky grains.

## Conclusion

In summary, we investigate the hcp/fcc nucleation in bcc iron under anisotropic compression with MD simulations. By adjusting the strain ratio along 3D axes, we obtain various shear deformations, covering from 1D to uniform and then to 2D compressions. Consequently, a significant influence of shear deformation on this structural transition is revealed. The pressure threshold roughly shows a linear change (16–32 GPa) with the extremum of shear stress ${\sigma }_{s}^{m}$ (−0.7–1.3 GPa) under those anisotropic compressions. Accordingly, the over-relaxation of shear stress and the increase of temperature and potential energy are all observed. As we know, there is still no systematic observations about the dependence of transition pressure on the shear deformation^39^. Obviously, these results are helpful to interpret the dispersion of the transition pressure^7,33^ in the lack of direct experimental observations.

Hcp/fcc nucleation processes are illustrated by the RDFs and microstructures. In the (001) 1D compression-dominated cases, only hcp nucleation occurs, which finally grows into larger hcp grains along the two invariant planes ((110) and (1–10)) under high strain rate. With reduction of the shear deformation, more equivalent planes are activated and also fcc nucleation can occur. In 2D compression-dominated cases, fcc nucleation will play a major role, and its mass fraction is already beyond hcp structure. Also, there are form many flaky grains and twins along the {110} planes. Both the hcp and fcc formations are the relative shuffle of adjacent {110} faces along the corresponding 〈110〉 directions, and the results depend on whether the shuffle directions are the same or the opposite. By comparing the variations of shear stress and potential energy of each phase, we further show the relaxation of shear stress for different phases and the energy relation of different phases, which further illustrate the important effect of shear stress on the hcp/fcc nucleation and growth. Obviously, these results can help us further understand the microscopic processes of bcc to hcp/fcc transition, and also their effects on the statistical properties, although these results are limited to the high strain rate (1.5 × 10^9^ s^−1^) and the specific loading path we set.
